# Genome sequence of *Billgrantia* sp. strain C5P2, isolated from the rhizosphere of *Brassica napus* grown in soda saline–alkaline soil

**DOI:** 10.1128/mra.01159-25

**Published:** 2026-03-27

**Authors:** Silva Lidice M. Perdomo, Wei Haozhuo, Qianqian Sun, Mohamed El Mahdi Ben Smaine, Tao Xinyi, Xing Qinghua, Belsti Atnkut, Zhao Baisuo

**Affiliations:** 1 Graduate School, Chinese Academy of Agricultural Sciences 12661, Beijing, China; 2 Institute of Plant Protection, Chinese Academy of Agricultural Sciences 12661, Beijing, China; 3 Department of Biology, College of Natural and Computational Science, Injibara University 635142, Injibara, Ethiopia; Indiana University Bloomington, Bloomington, Indiana, USA

**Keywords:** *Billgrantia*, genome sequence, ANI and dDDH, comparative genomics, *Halotolerant rhizobacterium*, plant growth-promoting rhizobacteria, soda saline-alkaline soil

## Abstract

The genome sequence of *Billgrantia* sp. strain C5P2, isolated from the rhizosphere of *Brassica napus* in soda saline–alkaline soil, is presented. The 4.76-Mb genome and genome-wide comparative analyses of strain C5P2 suggest that it may represent a distinct species within the genus *Billgrantia*.

## ANNOUNCEMENT

Strain C5P2 was isolated from the rhizosphere of 20-day-old *Brassica napus* var. Huayouza 62 cultivated in a controlled pot experiment. Plants were grown in a soil mixture consisting of 70% soda saline–alkaline soil collected from the Songnen Plain (Heilongjiang Province, China) and 30% commercial substrate. Rhizosphere suspensions were serially diluted and plated onto alkaline Luria–Bertani (LB) agar supplemented with 8% (wt/vol) NaCl (pH 8.5). Plates were incubated aerobically at 30°C for 24–48 h, and distinct colonies were purified by repeated streaking. This genome sequencing was performed to characterize the genomic features and clarify the taxonomic position of C5P2 within the genus *Billgrantia*.

**Fig 1:**
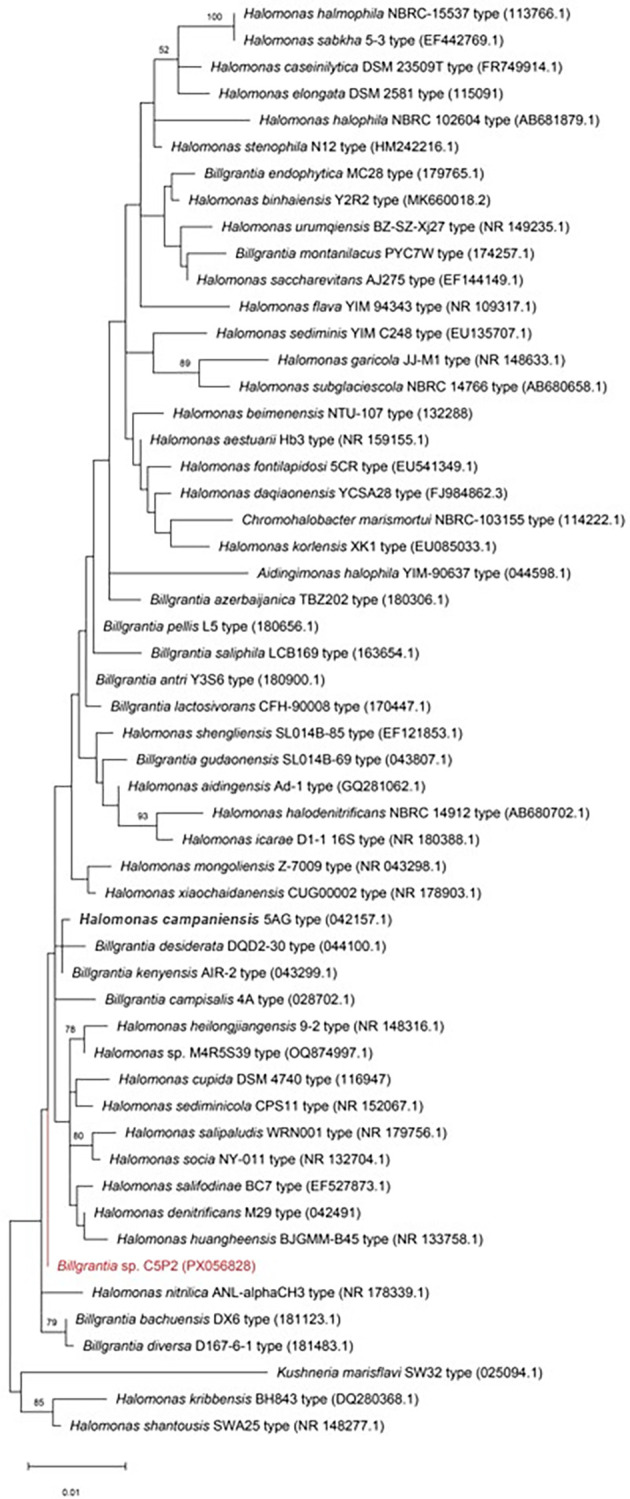
Maximum-likelihood phylogenetic tree based on 16S ribosomal RNA gene sequences showing the position of strain C5P2 within the family Halomonadaceae.

The nearly full-length 16S ribosomal RNA (rRNA) gene was PCR-amplified using universal bacterial primers 27F and 1492R (1) and sequenced by Sanger sequencing. The resulting sequence (PX056828.1; 1,375 bp) was compared against the EzBioCloud database (2) and showed highest similarity (98.47%) to *Billgrantia desiderata* strain FB2. Phylogenetic analysis was conducted using MUSCLE alignment in MEGA v12.0 (3), and a maximum-likelihood tree was inferred under the Tamura–Nei model with gamma-distributed rate variation and invariant sites (G+I) with 1,000 bootstrap replicates. Kushneria marisflavi SW32ᵀ served as the outgroup (Fig. 1).

For genomic DNA extraction, C5P2 was cultivated aerobically in LB medium containing 8% (wt/vol) NaCl (pH 8.5) at 30°C with shaking at 180 rpm. Cells from a 12-h culture were harvested by centrifugation (8,000 × *g*, 10 min), and genomic DNA was extracted using a magnetic-bead-based Bacterial/Fungal DNA Extraction Kit (Majorbio, Shanghai, China) following the manufacturer’s protocol.

An Illumina paired-end library (~455 bp insert size) was prepared using the NEBNext Ultra DNA Library Prep Kit (New England Biolabs) and sequenced on an Illumina HiSeq 4000 platform to generate 2 × 150 bp reads. A total of 8,056,216 raw paired reads were produced. Quality control was performed using fastp v0.20.0 (4) with default parameters, yielding 7,930,388 clean reads.

High-molecular-weight DNA was subjected to single-molecule real-time sequencing using PacBio HiFi chemistry on a Revio platform (Pacific Biosciences). DNA was sheared to 8–10 kb fragments using G-tubes (Covaris), and SMRTbell libraries were prepared with the SMRTbell Prep Kit 3.0 without additional size selection. Sequencing generated 33,404 subreads (369,263,678 bp) with a read N50 of 11,112 bp.

Hybrid genome assembly was performed using Unicycler v0.4.8 (5) with default parameters, and polishing was conducted with Pilon v1.22 (6) using Illumina reads mapped with BWA-MEM v0.7.17 (7). The final assembly consisted of a single circular chromosome of 4,762,915 bp with a guanine–cytosine (GC) content of 63.97% and an average sequencing depth of 328×. Circularization was confirmed by resolution of overlapping terminal regions during assembly. Completeness was assessed with BUSCO v5.4.5 (8) (bacteria_odb10; 98.4% complete, 1.6% fragmented) and CheckM v1.2.4 (9) (99.27% completeness; 0.82% contamination).

Genome-based relatedness was evaluated using average nucleotide identity (ANI) calculated with the ANI based on BLAST (ANIb) and ANI based on MUMmer (ANIm), algorithms implemented in JSpeciesWS (10) and digital DNA-DNA hybridization (dDDH) values calculated with genome-to-genome distance calculator v3.0 (formula 2) (11). The highest ANI values were observed with *Billgrantia bachuensis* (88.21%/89.38%) and *Billgrantia sulfidoxydans* (86.74%/87.91%), and corresponding dDDH values were 36.3% and 32.5%, respectively, all below accepted species thresholds. These results suggest that strain C5P2 may represent a distinct species within the genus *Billgrantia*.

Genome annotation using the National Center for Biotechnology Information (NCBI) Prokaryotic Genome Annotation Pipeline (v6.10) (12) identified 4,438 genes, including 4,336 protein-coding sequences. Secondary metabolite biosynthetic gene clusters were predicted using antiSMASH v6.1.1 (13), including siderophore-associated clusters.

## Data Availability

The genome sequence has been deposited in DDBJ/ENA/GenBank under accession number GCA_051062895.2. The corresponding RefSeq assembly is available under accession number GCF_051062895.2. The BioProject accession number is PRJNA1273590, and the BioSample accession number is SAMN48949116. Raw sequencing reads have been deposited in the NCBI Sequence Read Archive under accession numbers SRR35673024 and SRR35673023. The strain has been deposited in the China General Microbiological Culture Collection Center under accession number CGMCC 1.65540.

## AFTER EPUB

*[This article was published on 27 March 2026 with an error in an author affiliation.The author affiliation was corrected in the current version, posted on 29 April 2026.]*
